# Abstracts from Foreign Journals

**Published:** 1902-05

**Authors:** S. J. J. Harger

**Affiliations:** Philadelphia, Pa.


### ABSTRACTS FROM FOREIGN JOURNALS.

### FRENCH.

UNDER THE DIRECTION OF S. J. J. HARGER, V.M.D.,
PHILADELPHIA, PA.

PULMONARY NODES IN THE LUNG SIMULATING GLANDERS IN A HORSE AFFECTED WITH SUMMER WOUNDS. Summer wounds contain firm, nodular granulations of a grayish color that can be easily enucleated from the sclerosed fibrous tissue into which the germ is transformed. The centre of these nodes consists of a caseous amorphous material that contains the larva of a nematode.

Nocard observed some of these nodular growths in the lungs of a horse affected with an old summer wound. They are composed of a very thick, fibrous capsule, in the interior of which exists a small, grayish mass, glistening on section, easily enucleated, and leaving a small, smooth cavity. The contents are firm, but do not show any calcareous infiltration, and can be easily differentiated from glanders tubercles. A microscopical examination revealed the presence of a nematode larva identical with that found in the cutaneous nodosities.—*Annal de Med. Vet.*

COCAINE-MORPHINE ANALGESIA. Arloing, in 1885, demonstrated that the physiological effects of cocaine are due to alterations of the physical properties of the nerve fibrils and their terminals. Morphine acts in the same manner, but its action is of longer duration. Pecus, therefore, combines these two alkaloids in order to intensify and prolong the anæsthesia for diagnostic purposes. He supplies cocaine in a 3 per cent. and morphine in a 4 per cent. solution.

A therapeutic value has been ascribed to such an injection, but this is scarcely admissible.—*Annal de Med. Vet.*

CLYSTERS OF HYDROGEN PEROXIDE. Rager has practised washing out of the intestines for a long time. The commercial preparation being slightly acid, the following solution is used: 100 c.c. of hydrogen peroxide in 900 c.c. boiled water, to which is added 5 grains sodium chloride, 3 grains sodium phosphate, and 5 grains sodium bicarbonate. This solution is slightly alkaline. The object is to wash out the large intestine. This treatment has given excellent results in dysenteriform and muco-membranous colitis, as well as in divers other intestinal affections. Rocaz has employed it very successfully in acute dysentery in infants. Since in the dog we meet analogous intestinal disorder, this local method of medication should be of advantage in canine practice.—*Presse Medicale.*

Avian Diphtheria. Guérin, with many other observers, has been able to demonstrate that diphtheria in the fowl is caused by a microbe different from the bacillus of diphtheria of man. The former belongs to the pasteurella of Lignierès.

Guérin has experimented with vaccination against diphtheria in birds. Intra-peritoneal inoculation of cultures heated to divers degrees have given immunity to animals which have afterward resisted an inoculation with a virulent culture that was constantly fatal to control animals.

A preventive serum was also prepared. A horse which was injected repeatedly with virulent cultures furnished at the end of several months a serum capable of immunizing the pigeon, the most susceptible animal, against a virulent intra-peritoneal inoculation. The minimum dose of the serum was 4 c.c.; against a subcutaneous inoculation 2 c.c. sufficed. The immunizing effect of this serum can be prolonged by the intra-peritoneal injection of a culture twenty-four hours afterward—the same sero-vaccination as practised in erysipelas and cholera of the hog. The therapeutic value of the serum has been only very feeble.—*Annals de Med. Vet.*

Cutaneous Lymphadenoma in a Cow. Lienaux observed in a cow three years old and very cachectic, numerous tumors all over the cutaneous surface excepting at the inferior part of the members. The tumors were 4-5 cm. in diameter, flat, circular, slightly elevated, not hard, and somewhat elastic; this surface was smooth and deprived of hair, not ulcerated, and the epidermis normal, and over some of the tumors scaly without any inflammatory symptoms.

The nature of these tumors it would have been difficult to determine but for a tumefaction of the retropharyngeal, sublingual prescapular, precrural and supramammary lymphatic glands. This suggested the condition as being lymphadenoma, which was confirmed by a histological examination of the tumors, lymph glands, and the blood. The internal organs were normal.

Cutaneous lymphadenoma is common in man, but in veterinary pathology is exceedingly rare.—*Rec. de Med. Vet.*

The Cacodylates in Malarial Fever. The cacodylates, which are very effective in cachectic diseases, are not well tolerated by the digestive tract. According to the studies of Gautier, hypodermic injections of cocodylate of sodium—methylarsenate of sodium—is inoffensive to the stomach and is much more efficacious than quinine against malaria.

Repeated excesses of this disease, in spite of the administration of doses of quinine, were speedily relieved by this preparation in 5 cm. doses given hypodermically. The hematozoa which exist in abundance in the blood are rapidly destroyed by the mononuclear leucocytes. The latter are very much increased in number under the influence of this remedy.—*C. R. de l'Acad. des. Sciences.*

Insufflation of Atmospheric Air for Strained Tendons. Joly has employed the subcutaneous insufflation of atmospheric air to reduce inflammation of the tendons and their sheaths in strain of the perforans and perforatus.

The manner of operation is as follows: A Potain aspirator is used; the lateral needle is covered with several layers of iodoform gauze to filter the air, while the central needle is connected with an India-rubber tube whose end is provided with a sterilized hypodermic needle. The skin is disinfected with a rubber tourniquet applied to the forearm above the knee. The needle is inserted at the middle of the posterior region of the canon. The air is then pumped in and the subcutaneous tissue well filled, usually about six to eight syringefuls, and collodion applied over the puncture. The tourniquet is removed. The air which passes up into the forearm interferes but little with walking.

The results of the operation are regularly favorable. Every morning the air is massaged toward the diseased region. The crepitation disappears in from six to eight days, although the subcutaneous tissue remains emphysematous after the crepitation has disappeared. Besides the massage, cold water douches are employed.

About fifteen days after the operation the parts are almost normal and the inflammatory symptoms have generally disappeared and in three weeks after the application of the treatment the animal is ready to go to work.—*Rec. de Med. Vet.*

Fracture of the Glenoid Fibrocartilage of the Second Phalanx. The articular surface of the second phalanx is supplemented at its supraposterior border by a thick transverse pad of fibrocartilage which on each side gives attachments to the two terminal branches of the perforatus tendon. This fibrocartilage forms a part of the apparatus of suspension of the fetlock, and in going down hill or stopping suddenly a great deal of traction is applied upon it by the perforatus. Hence, it is sometimes, though rarely, torn away from the bone. The symptoms, among them lowering of the fetlock and turning up of the toe, are similar to those of fracture of the second phalanx.

The cause must be attributed to some alteration in cartilage, the the result of the constant traction or of a constitutional predisposition.

The diagnosis is difficult and must be differentiated from fracture of the second phalanx by the swelling, the absence of lateral displacement and of crepitation.

The prognosis is unfavorable and under ordinary conditions the animal should be destroyed.—*Rec. de Med. Vet.*

Treatment of Foot-and-mouth Disease. Jarré recommends in this disease the local application of a 33 per cent. solution of chemically pure chromic acid ; also in other aphthous affections of the buccal mucous membranes. The solution is escharotic, destroys the sensibility, and enables the animal to masticate food. One dressing generally suffices ; the fever subsides and the ulcers desiccate. Upon the feet the effect is analogous, although the dressing is repeated two or three times. In 1500 cases treated in France in this manner the ulcerations healed without any complications.—*Oesterr. Monatsch. für Thierheilk.*

Peroneal and Tibial Neurectomy in Spavin. Bayer operated on 8 cases which previously had been unserviceable ; all became serviceable, but 2 developed stringhalt. In 1, a trotter, the result was remarkable. Schimmel, Vogt, Nordheim, and Housen (7 cases) operated with good results.

Fröhner, from February 18, 1898, to August, 1899, operated upon 12 cases (oldest, one and a half years ; most recent, three months), with favorable results. Of 8 cases previously treated there is no comment on 6, but in 2 the canon and the scaphoid bones became fractured eight and four months afterward, respectively. These accidents were due to trophic changes that follow neurectomies in general.—*Monatsch. für Präk. Thierheilk.*

Operative Variation in Arytenectomy.—After reviewing the work of various operators, Hendrickx gives to this operation an affirmative value. Observing that in some instances the roaring became more intense after than before the operation, he made a post-mortem examination of such a larynx, and found that the lumen of the upper end of the trachea was obstructed by the curling inward of the severed ends of the first two tracheal rings. To obviate this in operating, tracheotomy is performed before the larynx is incised in the usual manner, but not through the first two tracheal rings. The trachea is at once plugged with iodoform gauze or cotton which replaces the tampon canula. This plug and the tracheal tube are removed after twenty-four hours. (The translator has found this procedure satisfactory.)—*Annals de Med. Vet.*

Situs Transversus in a Bull. Lohbeck, Chief Inspector at Abattoir of Cologne, reports an anomaly of this kind in a yearling bull—that the positions of the organs were reversed in relation to right and left. The aorta was on the right side of the median line; the right ventricle and right auricle were on the left side, and vice versa; the lungs were likewise transposed in their position.

The œsophagus was on the right side of the trachea. The abdominal visceræ were not examined, but it is believed that the "situs transversus" was complete. The animal was in a perfect state of health.—*Fl. u. Milchhyg.*

Barley as a Substitute for Oats in the Horse's Ration. Aruch and Lelli divided into two groups twenty-eight horses under similar conditions of age, weight, condition, and work. The first group was fed on barley. The second was the control group. Every horse was weighed twice daily. The experiment continued for forty-eight days and was divided in four periods of twelve days each; the last period was subdivided into two periods of six days each. Two kilos of barley were substituted for the same quantity of oats during each period until the substitution was complete. For each period the mean weight of each horse was determined, and at the end of the experiment the mean of the means was made.

Conclusions: The weight of these horses diminished as the barley was increased, but in very small proportion. Barley can be substituted, not only pound for pound, but completely without any bad effects upon the physical or dynamic conditions of the animal. This substitution has an economic value.—*Annals de Med. Vet.*

